# Single Port Laparoscopic Liver Resection for Hepatocellular Carcinoma: A Preliminary Report

**DOI:** 10.4061/2011/579203

**Published:** 2011-05-26

**Authors:** Stephen Kin Yong Chang, Maria Mayasari, Iyer Shridhar Ganpathi, Victor Lee Tswen Wen, Krishnakumar Madhavan

**Affiliations:** Division of Hepatobiliary and Pancreatic Surgery, National University Health System, NUHS Tower Block, Level 8, 1E Kent Ridge Road, Singapore 119228

## Abstract

Single port laparoscopic surgery is an emerging technique, now commonly used in cholecystectomy. The experience of using this technique in liver resection for hepatocellular carcinoma is described in a series of 3 cases with single port laparoscopic liver resection performed during 2010. All patients were male aged 61 to 70 years, with several comorbidities. There were no complications in this early series. The length of hospital stay was 3–5 days. The blood loss was 200–450 mL, with operating time between 142 and 171 minutes. We conclude that this technique is feasible and safe to perform in experienced centers.

## 1. Introduction

Laparoscopic liver resection has been increasingly performed over the last two decades. The technique has improved since the first time it was published [1] in 1992. Each year, there has been considerable number of cases undergoing this technique. Consecutive reports have shown that liver resection can be done efficiently and safely using laparoscopic approach. Several potential advantages include less abdominal pain, less hospital stay, and some reports [2, 3] even suggest less operating time with less morbidity. 

Single port laparoscopic surgery was reported as early as 1992 [4]. First described as an effort to reduce abdominal trauma in appendix removal, this approach has extended its indications to a variety of cases. One potential problem arising from this approach is the loss of triangular movement traditionally achieved with conventional laparoscopic surgery. In 2010, there were several publications [5–9] regarding single port laparoscopic liver resections. However these reports have limited coverage of this technique as a treatment for hepatocellular carcinoma (HCC). This study aims to report the feasibility and safety of single port laparoscopic liver resection technique for treatment of hepatocellular cancer in a single institution.

## 2. Methods

During 2010, all single port laparoscopic liver resection cases with proven histology findings of HCC were included in this paper. Demographic data, length of operation, operation technique, resection margin, blood loss, early post-op complications, and length of stay were evaluated for these patients. Postoperative followup was done until the end of 2010.

## 3. Surgical Technique

Patients were put under general anaesthesia in the French position. Incision was made according to the need to place the port. For GelPort (Applied Medical, Calif, USA), a 5 cm upper umbilical midline incision was made. For the SILS port (Covidien, Dublin, Ireland), a midline incision through the umbilicus measuring 2.5 cm was made. Port was inserted using open technique, and pneumoperitoneum of 12 mmHg was created using CO_2_. A 30° laparoscopic camera was used for visual inspection of the abdominal cavity. A bendable and roticulating instrument (AutoSuture Roticulator Endo Grasp from Covidien, Dublin, Ireland) was used to manipulate the liver, together with the normal laparoscopic instruments. Liver was mobilized from falciform ligament and left triangular ligament using harmonic scalpel and diathermy. Intraoperative ultrasound with laparoscopic ultrasound probe was done to assess the tumor, and margin of resection was marked using diathermy. Liver parenchyma was transected using Harmonic scalpel (Ethicon Endo-Surgery, Ohio, USA) and/or LigaSure (Covidien, Dublin, Ireland). Larger vascular structure such as the pedicle of liver segments and hepatic veins were divided using laparoscopic vascular stapler. Tissue glue was applied to the cut surface of liver. The specimen was retrieved using a plastic bag. Hemostasis was checked after desufflation of the abdomen. No drain was inserted at the end of the operation.

## 4. Results

In 2010, we have performed 3 cases of single port laparoscopic liver resection for HCC in our institution. All patients were male and had several comorbidities (Table 1). 

Case  1 was known to have non viral hepatitis cirrhosis likely secondary to non alcoholic steatohepatitis for 3 years, with a family history of liver cancer. He was found to have a nodule in segment 2 on the followup of the CT scan. Previously before the operation, patient was independent. He was Child-Pugh class A, and the Model (MELD for End-stage Liver Disease) score before operation was 8. Platelet count was normal.

Case  2 has been diagnosed with HCC previously and underwent laparoscopic liver resection twice for segment 5 and right posterior resection, respectively. Previous resections were 2 years and 6 months before the single port resection. Patient was ambulating independently, with MELD score of 6 and Child-Pugh class A status. Platelets count before operation was normal.

Case  3 presented with lesion in segment 2 liver, found during investigation for thrombocytopenia. Patient was also known to have fatty liver. MELD score before operation was 6. The total platelet count was in low borderline of 163 × 10^9^ /L. Patient was ambulating independently when admitted.

None of the operations were converted to open surgery. No additional port insertions were needed to complete the three operations. All patients stayed 1 night at the surgical high dependency unit and went to general ward the next day. Subsequent followup until December 2010 (7 months for case 1, 7 months for case 2, and 4 months for the last case) showed no recurrence of HCC. Detailed data on resection type, blood loss, operation duration, length of stay, complications, and resection margin can be seen in Table 2. There were no complications in this early series. The length of stay was 3–5 days. The blood loss was less than 500 mL in all cases. Operative time was less than 3 hours.

Although there is only one established cirrhosis for the nonneoplastic histopathology results for the resected specimen, case 2 shows occasional portal-portal fibrosis, and both cases without cirrhosis show portal chronic inflammation and macrovesicular steatosis.

## 5. Discussion

Laparoscopic liver surgery was firstly described by Gagner et al. [1] in 1992. Since that time, a number of studies [2, 3] regarding the feasibility and safety of the procedure have been published. During 2010, there have been several publications of the use of single port surgery for liver resection. The first report of this technique was by Aldrighetti et al. [5] in June 2010 who describes a left lateral sectionectomy for a single colorectal metastasis. The authors concluded that the approach is a feasible technique, but other benefits except cosmetic were questionable. 

 After the first report, several other publications reporting a single case or multiple case reports have been published. However, most of these cases were done for benign lesions or liver metastases. Only 1 case of single port liver resection from 5-case series reported by Gaujoux et al. [6] was done for hepatocellular carcinoma. Most reports for the single port laparoscopic liver resection were done for either benign lesions [6, 7] or metastatic lesions [5, 6, 8]

Single port laparoscopic liver resection is a new and emerging technique. With the development of special instruments to facilitate this technique, liver resection has become feasible and safe, but surgeons have been slow in applying this technique for HCC due to the presence of cirrhosis and concern regarding the oncological safety of the technique. The difficulty encountered when using single port laparoscopy is the loss of instrument triangulation, something that is crucial in a conventional laparoscopy. However, this setback can be overcome using new instruments with bending and angulating capability. Single port laparoscopy also requires the surgeon to do some cross-handling of the instruments that can facilitate the triangulation inside the abdominal cavity.

Starting in 2008, single port laparoscopic cholecystectomy has also been done regularly at our centre. Our centre's initial experience of single incision laparoscopic cholecystectomy [10] showed that there is no significant difference regarding pain and analgesia requirement between this technique and the conventional technique. Although there is still insufficient data to actually validate the clinical benefits of this technique over the conventional technique, the single port laparoscopy cholecystectomy feasibility is already established [11]. Our centre has begun offering the single incision laparoscopy cholecystectomy for patients on regular basis and has already exceeded 100 cases for the last 2 years.

In our centre, laparoscopic liver resections have been performed since 2005. Since then, more than 100 cases of laparoscopic liver resections have been done with good results. Combining this technique with the single port laparoscopic cholecystectomy experience in our centre, the single port liver resection was started in 2010.

Our experience shows that the single port laparoscopic liver resection approach can be done with reasonable operating time. Previous publications [5–7] reported that operative time for single port liver resection ranged from 55 minutes to 145 minutes. These results were comparable to our experience, with time range of 142–171 minutes. In our series, there is a slightly higher blood loss compared to other publications [5–7] (20–80 mL). All our patients were on anticoagulant therapy prior to their surgery, and this possibly explains the higher blood loss in our experience. The other reason for the difference was likely due to the size of the tumor and the underlying cirrhotic liver in one of our 3 patients, resulting in difficulties to achieve hemostasis. 

Left segmental/sectional resection of the liver has been the main type of resection for single port laparoscopic liver surgery. Patients with lesions limited to the left side of the liver are appropriate for this technique, as reported in our series. This type of resection is best suited for single port technique because the instruments are already aligned to the liver transection plane and the specimen is small enough to be retrieved through a small incision (less than 5 cm). Of all the published single port liver resection cases [5–9], none were converted to open surgery. The postoperative hospital stay was also shorter. Our experience in this small series has been the same. As for the resection margin for the specimen, the result showed that a considerable free margin can be achieved. This indicates that the technique is not only feasible but also safe to perform in experienced centers.

## 6. Conclusion

This early experience with single-port liver resection for HCC suggests that this operation is safe and feasible in selected cases of HCC in a unit with experience in laparoscopic liver resection and single-port surgery.

## Figures and Tables

**Table 1:** Patient comorbidities.

Case	Age	Gender	Comorbidities
1	61	Male	Ischemic heart disease with previous coronary artery bypass
			Hyperlipidaemia
			Type II diabetes mellitus

2	69	Male	Type II diabetes mellitus
			Hepatitis B carrier
			Ischemic heart disease
			Asthma
			Old cerebrovascular accident
			Hypertension

3	70	Male	Type II diabetes mellitus
			Hypertension
			Ischemic heart disease
			Beta thalassaemia trait
			Fatty liver
			Mild esophagitis
			Thrombocytopenia
			Renal calculi

**Table 2:** Operative parameters.

Case	Resection Type	Tumor size	Cirrhosis	Blood loss	Length of operation (min)	Length of stay	Complications	Resection margin
1	Left lateral sectionectomy	3.5 cm	+	450	171	4 days	Nil	2.5 cm
2	Segment 3 liver resection	2 cm	−	200	142	3 days	Nil	0.4 cm
3	Left lateral sectionectomy	4.5 cm	−	300	159	5 days	Nil	0.7 cm
